# Genomic selection for tolerance to aluminum toxicity in a synthetic population of upland rice

**DOI:** 10.1371/journal.pone.0307009

**Published:** 2024-08-22

**Authors:** Jérôme Bartholomé, José Omar Ospina, Mario Sandoval, Natalia Espinosa, Jairo Arcos, Yolima Ospina, Julien Frouin, Cédric Beartschi, Thaura Ghneim, Cécile Grenier

**Affiliations:** 1 CIRAD, UMR AGAP Institut, Montpellier, France; 2 UMR AGAP institut, Univ Montpellier, CIRAD, INRAE, Institut Agro, Montpellier, France; 3 Alliance Bioversity CIAT, Cali, Colombia; 4 FEDEARROZ–Fondo Nacional del Arroz, Bogotá, Colombia; 5 HarvestPlus Program, Alliance Bioversity CIAT, Cali, Colombia; 6 Departamento de Ciencias Biológicas, Universidad ICESI, Cali, Colombia; Government College University Faisalabad, PAKISTAN

## Abstract

Over half of the world’s arable land is acidic, which constrains cereal production. In South America, different rice-growing regions (Cerrado in Brazil and Llanos in Colombia and Venezuela) are particularly affected due to high aluminum toxicity levels. For this reason, efforts have been made to breed for tolerance to aluminum toxicity using synthetic populations. The breeding program of CIAT-CIRAD is a good example of the use of recurrent selection to increase productivity for the Llanos in Colombia. In this study, we evaluated the performance of genomic prediction models to optimize the breeding scheme by hastening the development of an improved synthetic population and elite lines. We characterized 334 families at the S_0:4_ generation in two conditions. One condition was the control, managed with liming, while the other had high aluminum toxicity. Four traits were considered: days to flowering (FL), plant height (PH), grain yield (YLD), and zinc concentration in the polished grain (ZN). The population presented a high tolerance to aluminum toxicity, with more than 72% of the families showing a higher yield under aluminum conditions. The performance of the families under the aluminum toxicity condition was predicted using four different models: a single-environment model and three multi-environment models. The multi-environment models differed in the way they integrated genotype-by-environment interactions. The best predictive abilities were achieved using multi-environment models: 0.67 for FL, 0.60 for PH, 0.53 for YLD, and 0.65 for ZN. The gain of multi-environment over single-environment models ranged from 71% for YLD to 430% for FL. The selection of the best-performing families based on multi-trait indices, including the four traits mentioned above, facilitated the identification of suitable families for recombination. This information will be used to develop a new cycle of recurrent selection through genomic selection.

## Introduction

Acid soils represent over 50% of the world’s arable land and are a significant constraint on cereal production [1]. Acidity is caused by low saturation of cations due to leaching triggered by intense weathering in a humid tropical climate. Most of these soils are present in the tropics, especially in South America and Africa, in a proportion of 43% and 27%, respectively (Okagawa 1984 in [2]). The production of most staple crops is negatively impacted by acid soils [3]. Aluminum (Al) is a metal naturally abundant in the soil, which, when pH values fall below 5.0, becomes highly phytotoxic by adopting a trivalent form (Al^3+^) [4]. Once solubilized, Al causes rapid root growth inhibition, leading to a reduced and stunted root system. Al^3+^ alters the ability of plants to acquire both water and nutrients. While rice (*Oryza sativa*) is known to be one of the most tolerant species among the Poaceae [5], the level of tolerance varies greatly within the species, with *japonica* accessions being, on average, more tolerant than *indica* accessions [6]. A large fraction of the acid soils in the southern tropical belt consisting of ultisols and oxisols are vast areas of anthropic savannas on heavily eroded and degraded soils [7]. In South America, acid soils support sub-humid savannas (Cerrados in Brazil and Llanos in Colombia and Venezuela) and the tropical rainforest of the Amazon basin [2]. Because of its relatively good tolerance to acid soils, upland rice is an important component of rice-pasture cropping systems, developed as both productive and sustainable for the savannas [2].

Many studies have focused on the response to Al toxicity to identify tolerant lines and improve productivity [8–11]. More recently, the efforts were focused on the analysis of morpho-physiological traits to characterize the genetic control of mechanisms involved in crop tolerance to high Al^3+^ concentrations [6, 12, 13]. In this context, tolerance to Al toxicity was assessed primarily by examining root and shoot characteristics during the vegetative phase under hydroponic conditions. While the mechanism of root tip exclusion of Al^3+^ appears as a primary control of tolerance to Al toxicity, scientific reports often highlight the complexity of tolerance to Al toxicity [14–17]. Numerous QTLs for rice tolerance to Al toxicity have been described, and several genes have been cloned [18–20]. Therefore, the genetic regulation of rice’s ability to withstand Al toxicity is intricate and involves multiple genes with varying degrees of effect. Improving tolerance to Al toxicity in rice thus translates into manipulating a quantitative trait that can be bred for with an appropriate breeding scheme.

Today, one of the most efficient molecular breeding approaches to improve traits controlled by many genes is undoubtedly genomic selection (GS). Through the integration of molecular markers covering the whole genome, GS is used to estimate the breeding value of selection candidates. First proposed in 2001 for animal breeding [21], GS was first evaluated in plant species in the late 2000s [22–25]. Empirical and simulation studies have confirmed the benefits of GS since then [26, 27]. Best adapted to polygenic traits such as yield, GS has been applied in rice since 2014 (see [28, 29] for reviews). As for phenotypic selection, a central component of GS in plant breeding is the integration of genotype by environment interactions (GxE) [27, 30]. Since 2012, several prediction models taking into account GxE have been proposed [31]. First, Burgueño et al. [32] extended the single-environment genomic best linear unbiased prediction (GBLUP) to incorporate multiple environments. This approach has been further developed in various studies with different statistical methods, such as non-linear kernels and Bayesian inference [33–36]. In parallel, reaction norm models taking advantage of environmental covariates were also developed to better capture GxE [37, 38]. These recent advancements in GS have improved data integration from multi-environment trials, leading to increased accuracy in most cases [31]. One approach to predicting adaptive traits or tolerance to abiotic stresses is to include a GxE interaction component in the GS model, which is a common practice according to previous studies [29]. Another option is to create a two-step model based on an index used to express the tolerance of the genotypes to the constraining factor under consideration [39].

Rice breeding for tolerance to acid soils began in the CIAT in the early 1980s to develop upland rice lines well-suited to the soil conditions found in savannas. The goal was to establish more stable and productive rice-pasture cropping systems in these environments. The breeding program aimed to develop rice plants that could grow in acidic, nutrient-poor soil with high levels of Al saturation with early vigor, resistance to rice blast (*Pyricularia grisea* Sacc.), good grain quality (translucent, long-slender grain), and early maturity (total cycle about 115 days) [9, 10]. First, a large collection of material was screened for its adaptation to acid soils [8]. The best material was used as parents for crosses in a classical pedigree breeding approach. From these efforts, a variety derived from a cross between *indica* and *japonica* rice was released by CIAT and ICA (Colombian Agricultural Institute) in 1991 as "Oryzica sabana 6" [40]. It combined the productivity of the indica and the tolerance to Al toxicity of the tropical japonica. In parallel to pedigree breeding, recurrent selection based on a synthetic population was used to develop improved material with a particular focus on adaptation to acid soils [41]. A large base population was first developed with the contribution of 27 rice varieties from Brazil, Africa, and Asia selected for their adaptation to upland cultivation crossed with a male sterile rice cultivar (IR36) [42]. This initial pool of germplasm was recombined for several cycles and further improved with successive additions of 21 upland elite lines from CIRAD (French Agricultural Research Centre for International Development), IRRI (International Rice Research Institute), and Embrapa (Brazilian Agricultural Research Corporation) [43, 44]. The resulting populations improved through recurrent selection and formed a diverse pool of genotypes with an increased frequency of favorable alleles to be further exploited through pedigree breeding. Recurrent selection has the advantage of targeting the improvement of quantitative traits with a large number of loci involved, which is usually the case for adaptive traits. Several cycles of recurrent selection on acid soils with Al toxicity have resulted in a population with a high potential for productivity in the Llanos of Colombia [45].

Recently, the program has explored the potential of early genomic prediction to decrease cycle time and increase selection intensity. In this study, we evaluated the performance of genomic prediction models trained on S_0_-derived families to enhance the tolerance to Al toxicity of the upland rice synthetic population. We evaluated the population under two conditions: acid soil with Al toxicity and control managed with liming. The yield stability index was computed and also predicted to identify tolerant families. In addition, the performance of the families under Al toxicity was predicted using three multi-environment models (including GxE or not). Using estimated values derived from the best-performing model, we developed a multi-trait selection index that included the yield stability index. Finally, the opportunity to optimize the CIAT-CIRAD breeding scheme by hastening the development of an improved population and identifying promising elite lines was discussed.

## Material and methods

### Plant material

The synthetic population (PCT27) used in this study is from the tropical *japonica* group of rice (*Oryza sativa* L.). Synthetic populations are developed by crossing multiple genotypes, either segregating or fixed, known for their agronomic performance. In the case of the PCT27, the population was derived from previous synthetic populations of the CIAT-CIRAD breeding program to improve the Al tolerance of upland rice via recurrent selection using genic male sterility (ms) [45, 46]. The PCT27 population was formed from advanced lines derived from four original populations. These advanced lines were thirty-five S_2:4_ families represented by one fertile plant heterozygous for the *ms* gene [ms:MS] identified using marker-assisted selection [47]. The PCT27 was then generated by mixing equal amounts of seeds from each selfed plant. Two cycles of recombination were then performed using male-sterile plants, as the *ms* gene is segregating in the population. A subset of the population (334 S_0_) was selected to evaluate the performance of genomic prediction models [48]. The S_0_ were genotyped and then advanced at the S_0:4_ generation (four generations of inbreeding with bulk harvest) for phenotyping.

Six checks were also used to assess the response to Al toxicity. Those were the tropical *japonica* variety Azucena, the *indica* mega variety IR64, and four elite *indica* cultivars released by FEDEARROZ (Fondo Nacional del Arroz) for their high productivity achieved under favorable rainfed conditions (FED67, FED68, FED70, FED_Ibis).

### Field trial and phenotyping

Field phenotyping was performed during the main rice season in Colombia in 2020 at the FEDEARROZ experimental station in Santa Marta Aguazul, in the Casanare department of Colombia (4°59’38.23"N; - 72° 23’59.55"O, and 290 masl). We compared two conditions on the same field, characterized by its acid soil. On one part of the field, liming with CaCO_3_ and MgCO_3_ was applied in a ratio Ca:Mg = 4:1 to correct for soil acidity three months before the sowing by broadcast and vertical incorporation with vibratory chisels at a depth of 0–20 cm. This condition is referred to as LIM in the rest of the article. On the other half of the field, no treatment was applied (hereafter referred to as ALU). The two conditions were set 20 meters apart. Soil texture and chemical analysis are provided in Table 1.

**Table 1:** Soil chemical characteristics for the two conditions: LIM with liming three months before sowing and ALU without liming.

	pH	O.M.	P	Ca	Mg	K	Al	Al Sat
		g.kg^-1^	mg.dm^-3^	cmol.dm^-3^				%
	LIM							
**Mean**	5.19	1.82	15.84	5.00	1.60	0.43	0.53	7.35
**sd**	0.35	0.23	2.93	1.24	0.33	0.08	0.38	5.96
	ALU							
**Mean**	4.39	1.71	20.32	2.07	0.55	0.38	2.32	35.17
**sd**	0.04	0.15	7.34	0.73	0.13	0.03	0.33	5.85

The average value was obtained from six samples taken at 0-20cm depth in each field. The quantification of organic mater (O.M.) and different elements (phosphorus (P), calcium (Ca), magnesium (Mg), potassium (K), and Aluminum (Al)) is provided. Aluminum saturation (Al Sat) is calculated as the ratio between the exchangeable Al and the effective cation exchange capacity.

For each condition, the trial followed a partially replicated experimental design (p-rep) to distribute 334 genotypes and six checks in 36 blocks of 14 plots. The percentage replication in the p-rep was 25% of the PCT27, and each check was repeated six times. The trial was established by direct seeding. The plot size was two rows of 3 meters with 26 cm between rows. For both conditions, no additional irrigation was applied to the plots. The fertilizer application was split into three, with NPK nutrients (230 kg/ha urea, 217 kg/ha DAP, 150 kg/ha KC) added 8, 20, and 30 days after sowing. No phytosanitary treatment was applied.

Four traits were measured following the IRRI Standard Evaluation System [49] on all the material included. Flowering date (FL) was expressed as the number of days after sowing when 50% of the plants within a plot reached anthesis. Plant height (PH) was calculated as the average height measured in centimeters of five plants with their panicle extended. Grain yield (YLD) was obtained by weighing the grains collected within each plot and expressed in grams per plot for a relative humidity of 14%. The grain zinc concentration (ZN), expressed in parts per million (ppm), was measured on a sample of harvested grains, polished in Teflon equipment, and using energy dispersive X-ray fluorescence spectrometry (X-supreme 8000, Oxford Instrument, Shanghai, CN) available at the CIAT-HQ Nutritional Laboratory. The yield stability index (iYLD) was calculated as $iYLD=\frac{{{YLD}_{ALU}-YLD}_{LIM}}{{YLD}_{LIM}}*100$.

### Genotyping

Genotyping-by-sequencing was performed on the 334 S_0_ plants as described in Baertschi et al. [48].

Briefly, the fastq sequences were aligned to the reference genome, Os-Nipponbare-Reference-IRGSP-1.0, using Bowtie2. SNP calling was performed using the Tassel GBS pipeline [50]. Loci were filtered based on several criteria: missing data (<20%), depth for each data point (>10), minor allele frequency (>2.5%), and bi-allelic status of SNPs. To ensure accuracy, the read depth for SNP calling was set to a minimum of 10 to minimize the probability of under-calling a heterozygous site. Missing data were then imputed using Beagle 4.1. After quality control, 9928 SNPs remained for the genetic characterization (S1 Fig). The population analyzed in this study was not depleted of rare alleles, as evidenced by the minor allele frequency distribution (S2 Fig). Although the degree of allelic fixation varied considerably among the genotypes, individuals in the S_0_ generation exhibited relatively low levels of fixation. The average marker density of 1 SNP every 40 kb was sufficient to capture all linked QTLs with the SNP matrix, considering the relatively large average linkage disequilibrium (LD) and the slow LD decay observed (S3 Fig). Furthermore, no genetic structure was identified among the set of genotypes (S4 Fig).

### Statistical analyses

#### Phenotypic analysis

The single-trial analysis of variance was performed using the *SpATS* function of the R package *SpATS* [51]. We used a mixed model integrating two-dimensional P-splines to adjust for spatial heterogeneity [52, 53] using plot coordinates: row and column. For each condition, the following model was used:

$$y=X\beta +Z_{u}s+X_{s}\beta_{s}+Z_{s}s+\varepsilon .$$

where *y* is the vector of phenotypes, *β* is the vector of fixed effects, including the intercept and genotypes (families and checks), and *X* is the associated design matrix. The vector *u* includes mutually independent sub-vectors of random row (r) and column (c) effects that account for discontinuous variation. The design matrix *Z*_*u*_ = [Zr|Zc] and covariance matrix $U=diag(\sigma_{r}^{2}I_{r},\sigma_{c}^{2}I_{c})$ are used to model these effects. The smooth spatial surface is expressed as a mixed model comprising the fixed term *X*_*s*_*β*_*s*_ and the random term *Z*_*s*_*s*, where *s* is a vector of random spatial effects with a covariance matrix *S*. The design matrices *X*_*s*_ and *Z*_*s*_ and the covariance matrix *S* have been described previously [53]. The vector ε represents the residuals (also called nugget) with distribution $\varepsilon ∼N(0,\sigma_{\varepsilon }^{2}I)$. A similar model was used to estimate the variance components with the only difference that the genotypes were treated as a random effect. Broad sense heritability (H^2^) was estimated per condition using the following equation:

$$H^{2}=\frac{\sigma_{G}^{2}}{\sigma_{G}^{2}+\sigma_{\varepsilon }^{2}}$$

where $\sigma_{G}^{2}$ is the genetic variance of the trait under study and $\sigma_{\varepsilon }^{2}$ is the variance of the residuals. The phenotypic performances for subsequent analyses were represented by the best linear unbiased estimators (BLUEs) for each trait. The correlations of phenotypic performances (BLUEs) between the two conditions were performed using the *rcorr* function of the R package *Hmisc* [54]. Multivariate analysis was conducted with the R package *Factominer* [55].

#### Genomic selection

We used GS models to predict the performance of the S_0_ families under ALU. We compared two cross-validation (CV) scenarios. For each scenario, the population was divided into a number of folds (k) of approximately equal size. The first scenario (single-environment) was set to estimate the predictive ability of the model when only the ALU condition was used. In this scenario, the data in k-1 folds was used to train the model and predict the phenotypes in the remaining fold (the testing fold). The second scenario (multi-environment) took advantage of the information on the two conditions (LIM and ALU). The training set was composed of the whole population phenotyped in LIM and k-1 folds phenotyped in ALU. The testing set comprised the remaining fold of the families phenotyped in ALU. For the two scenarios, a 5-fold CV was used with 80% of the population assigned to the training set and 20% to the testing set in each successive fold, and ten repetitions were performed. The predictive ability was estimated as the Pearson correlation between genomic estimated breeding values (GEBV) and BLUEs of the validation set.

The Genomic Best Linear Unbiased Predictor (GBLUP) was used for multi-environment models [56]. The prediction design was similar to a sparse testing scheme where some lines were evaluated in some environments but not in others [57]. All predictions were performed using the R package *BGGE*, which allows different integration of multi-environment information and the modeling of GxE interactions [58, 59]. For the first scenario, single-environment (SM) GBLUP was performed, and we predicted the performance under ALU and LIM. For the second scenario, three different multi-environment models were implemented, all used to predict the performance under ALU: i) a multi-environment model (MM) assuming that genetic effects across the environment are constant, and therefore the absence of GxE interactions; ii) a multi-environment model (MDs), which is an extension of the MM model including a single random deviation effect of the GxE; and iii) a multi-environment model (MDe) with an environment-specific variance deviation effect for the GxE. More details about these models can be found in [58]. All four models were performed using the following parameters: burn-in = 5,000, nIter = 70,000, and thin = 5. The genomic relationship matrix was estimated using the linear kernel with the *getK* function from *BGGE*.

For each trait and model, variance components and GEBVs were obtained. Predictive abilities (PAs) were estimated as the correlation between the GEBVs and the BLUEs in ALU. The PA between the models was compared with Tukey’s range test.

#### Selection of the families

Our objective was to propose a selection for the families based on a linear GS index (GSi) that combined the four traits predicted for the ALU condition with the MM model (FL, PH, YLD, and ZN). We compared it to a phenotypic selection index (PSi) based on the same four traits observed in the LIM condition combined with the yield stability index (iYLD). The selection indices had the following general form: *I*_*G*_ = *β*′*γ*, where β is the unknown vector of weights and γ the vector of genomic estimated breeding values or phenotypic values [60]. β was estimated with the following equation: $\hat{\beta }=P^{-1}Gw$, where *P* and *G* are the variance-covariance matrices of phenotypic and genetic values, respectively, and *w* the vector of economic weights [61, 62]. In the GSi case, the matrix *P* was calculated using the BLUEs, while the matrix *G* was computed using the GEBVs predicted by the model MM. On the other hand, for PSi, the matrix *P* was estimated using BLUEs in LIM, and the matrix *G* was estimated using the GEBV generated by the SM model for the LIM condition without cross-validation. The weights (*w*) were defined based on empirical observations from previous RS cycles: 0, -0.2, 1, and 0.8 for FL, PH, YLD, and ZN, respectively. For the PSi, the iYLD was given a weight of 0.5.

The top 10% of the families were selected based on the indices, and the selection differential (S) was calculated as the difference between the mean of the whole population and the mean of the selected families for a given trait.

## Results

### Phenotypic variability in relation to aluminum phytodisponibility

**Fig 1:**
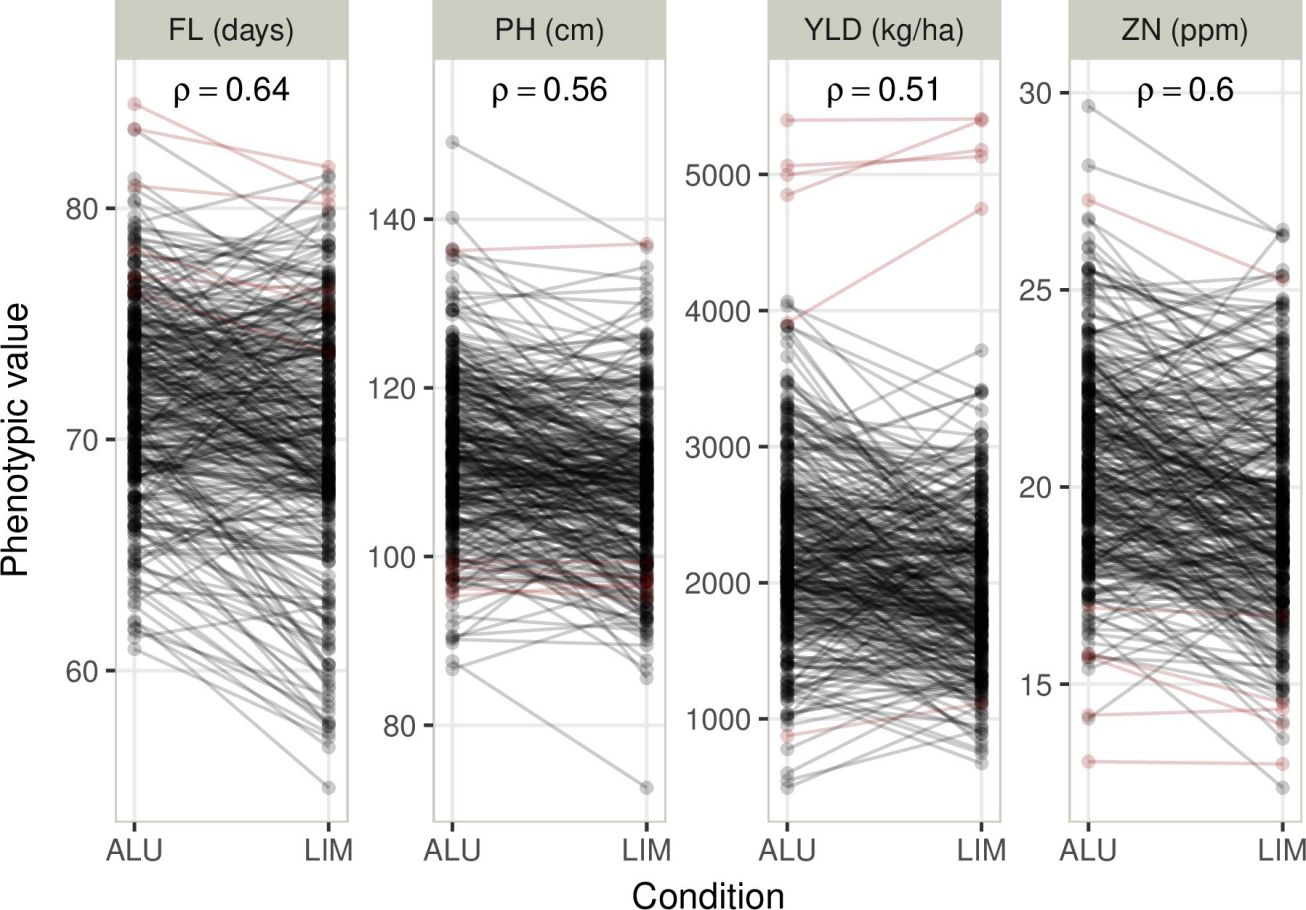
Distribution of the four traits evaluated in two conditions: Flowering time (FL), plant height (PH), grain yield (YLD), and zinc concentration in polished grain (ZN). The population is in black, and the controls are in red. The correlation coefficients (Spearman ρ) between the two conditions (ALU and LIM) for the population are indicated at the top of each panel.

Broad sense heritabilities measured within conditions (Table 2) were moderate (0.56) to high (0.75), indicating limited spatial variations in both experiments. The highest values were obtained for FL (0.75 in ALU and 0.74 in LIM) and the lowest for PH (0.6 in ALU and 0.56 in LIM). For all the traits except YLD, heritabilities were lower in LIM compared to ALU. The reduction of Al phytodisponibility in LIM compared to ALU had a limited impact on the average phenotypic performance of the population. The population flowered earlier in LIM (70.2 days) than in ALU (71.8). We observed a reduction of 4 cm for PH and 1.2 ppm for ZN in LIM compared to ALU (Table 2). Interestingly, YLD increased, on average, from 1941 kg/ha in LIM to 2219 kg/ha in ALU. The opposite trend was found for the checks with higher YLD in LIM compared to ALU, confirming the tolerance to Al toxicity of the population (Fig 1). The soil conditions also impacted the phenotypic variability associated with the average performance of the population. For most of the traits, CVs were lower in ALU compared to LIM (Table 2). Among the four traits, FL and PH were the least variable, followed by ZN, with values ranging from 5.7% for FL in ALU to 13% for ZN in LIM. As expected, YLD was the trait for which the CVs were the highest: 28.9% (ALU) and 28.8% (LIM). A moderate GxE interaction was found between the two conditions. Indeed, the correlations between conditions were all significant (p<0.05), ranging from 0.51 for YLD to 0.64 for FL (Fig 1). In both conditions, the correlations between FL and YLD, were negative: -0.39 and -0.29 for ALU and LIM, respectively (S5 Fig). Significant but lower correlations were also found between ZN and YLD: -0.16 and -0.20 for ALU and LIM, respectively.

**Table 2:** Descriptive statistics for the four traits considered: Days to flowering (FL, days), plant height (PH, cm), grain yield (YLD, kg/ha), and grain zinc concentration (ZN, ppm). In addition to the mean and range, the coefficient of variation (CV) and broad-sense heritability (H^2^) are presented.

TRAIT	Condition	Mean*	Range	CV	H^2^
**FL**	ALU	71.8 ^a^	60.9–83.4	5.69	0.75
	LIM	70.2 ^b^	54.9–81.4	7.25	0.74
**PH**	ALU	112 ^a^	86.7–149	8.28	0.6
	LIM	108 ^b^	72.6–137	8.65	0.56
**YLD**	ALU	2219 ^a^	494–4063	28.9	0.65
	LIM	1946 ^b^	674–3707	28.8	0.71
**ZN**	ALU	20.6 ^a^	14.1–29.7	11.8	0.7
	LIM	19.4 ^b^	12.4–26.5	13	0.65

* Within a trait, means followed by different letters are significantly different at p<0.05

### Evaluation and prediction of tolerance to aluminum toxicity

Based on the yield stability index iYLD, the population presented good tolerance to Al toxicity, with 72% of the families being tolerant (iYLD > 0). The average iYLD value for the population was 19.1. This masked a large variation between families, with index values ranging from -56.8 to 151.0. iYLD was significantly correlated with PH in LIM (-0.28, p < 0.01) (S5 Fig). Interestingly, this negative phenotypic correlation between iYLD and PH was not found in ALU conditions.

**Fig 2:**
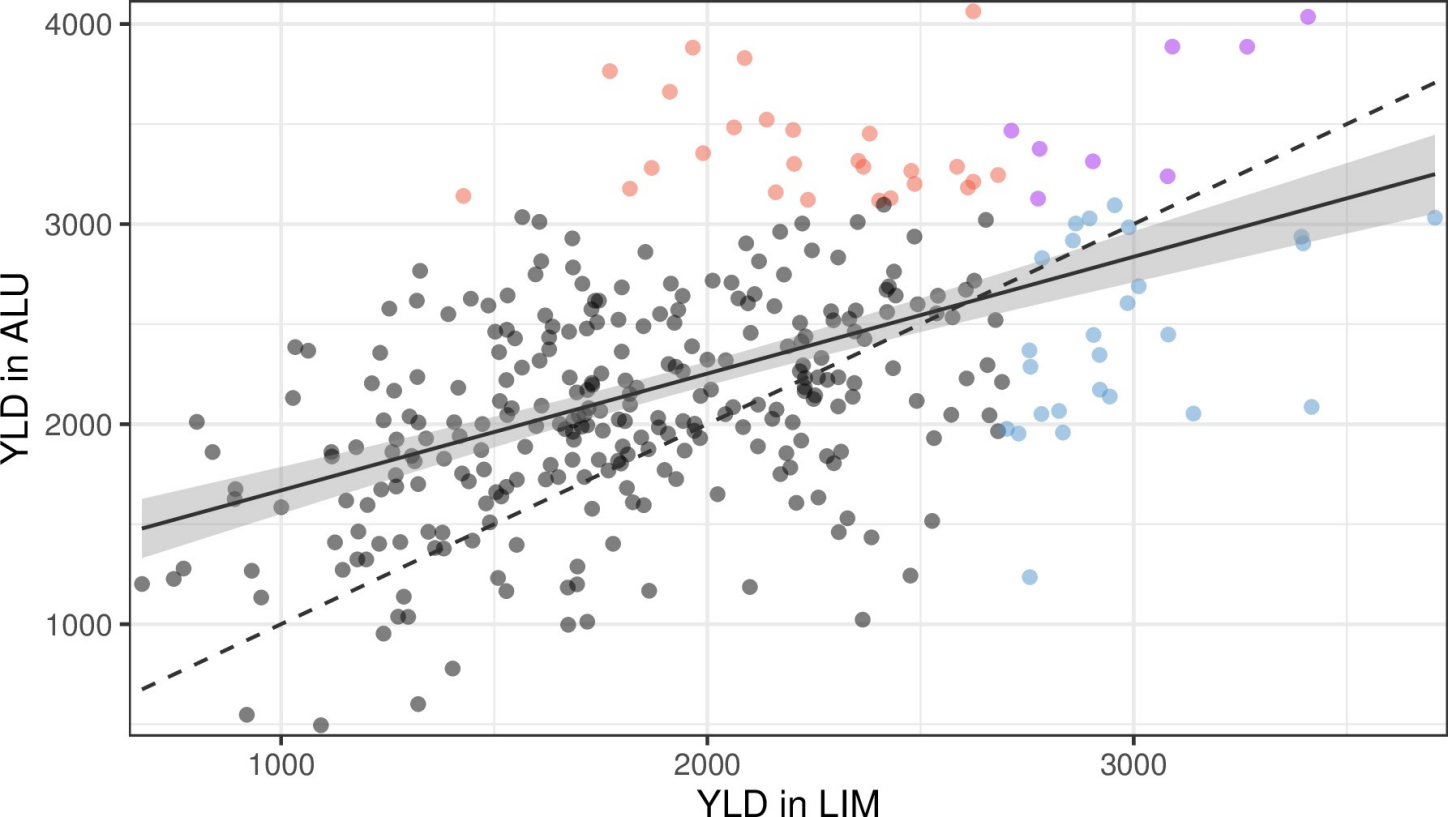
Identification of the best-performing families for grain yield (YLD) in the two conditions (LIM and ALU). The best ten percent of families in LIM or ALU are represented in blue and red, respectively. In purple are the best families in the two conditions. The dashed line represents the equation y = x, and the solid line represents the linear regression between the two conditions with the 95% confidence interval (grey area).

The high-yielding families were identified based on the population distribution for productivity under the two soil conditions (Fig 2). Only eight families were considered to be high-yielding in the two conditions. The high-yielding families displayed different behaviors in terms of relative performance (iYLD). As expected, the families with the highest yield in ALU were, on average, more tolerant than the rest of the population (iYLD = 46.7). On the contrary, the families performing better under LIM had, on average, an iYLD of -9.3.

The SM model’s genomic prediction for iYLD resulted in a very low PA of -0.12, as shown in Table 3. This is much lower than the estimated value for YLD in the ALU or LIM condition (S1 Table). However,

the prediction for iYLD via the ratio of predicted yield values in the two conditions was also poorly correlated with iYLD (-0.13) (S6 Fig).

**Table 3:** Predictive abilities (mean and standard deviation (SD)) obtained with the four genomic prediction models (see material and methods). The four traits are presented: days to flowering (FL), plant height (PH), grain yield (YLD), grain zinc concentration (ZN), and the index of yield stability under aluminum toxicity (iYLD).

Trait	METHOD	MEAN	SD
**FL**	SM	0.126	0.035
	MM	0.668	0.002
	MDs	0.659	0.004
	MDe	0.634	0.006
**PH**	SM	0.324	0.023
	MM	0.603	0.002
	MDs	0.585	0.009
	MDe	0.563	0.013
**YLD**	SM	0.258	0.031
	MM	0.531	0.003
	MDs	0.476	0.012
	MDe	0.442	0.015
**ZN**	SM	0.335	0.031
	MM	0.647	0.002
	MDs	0.612	0.009
	MDe	0.587	0.013
**iYLD**	SM	-0.117	0.024

### Performance of multi-environment models

**Fig 3:**
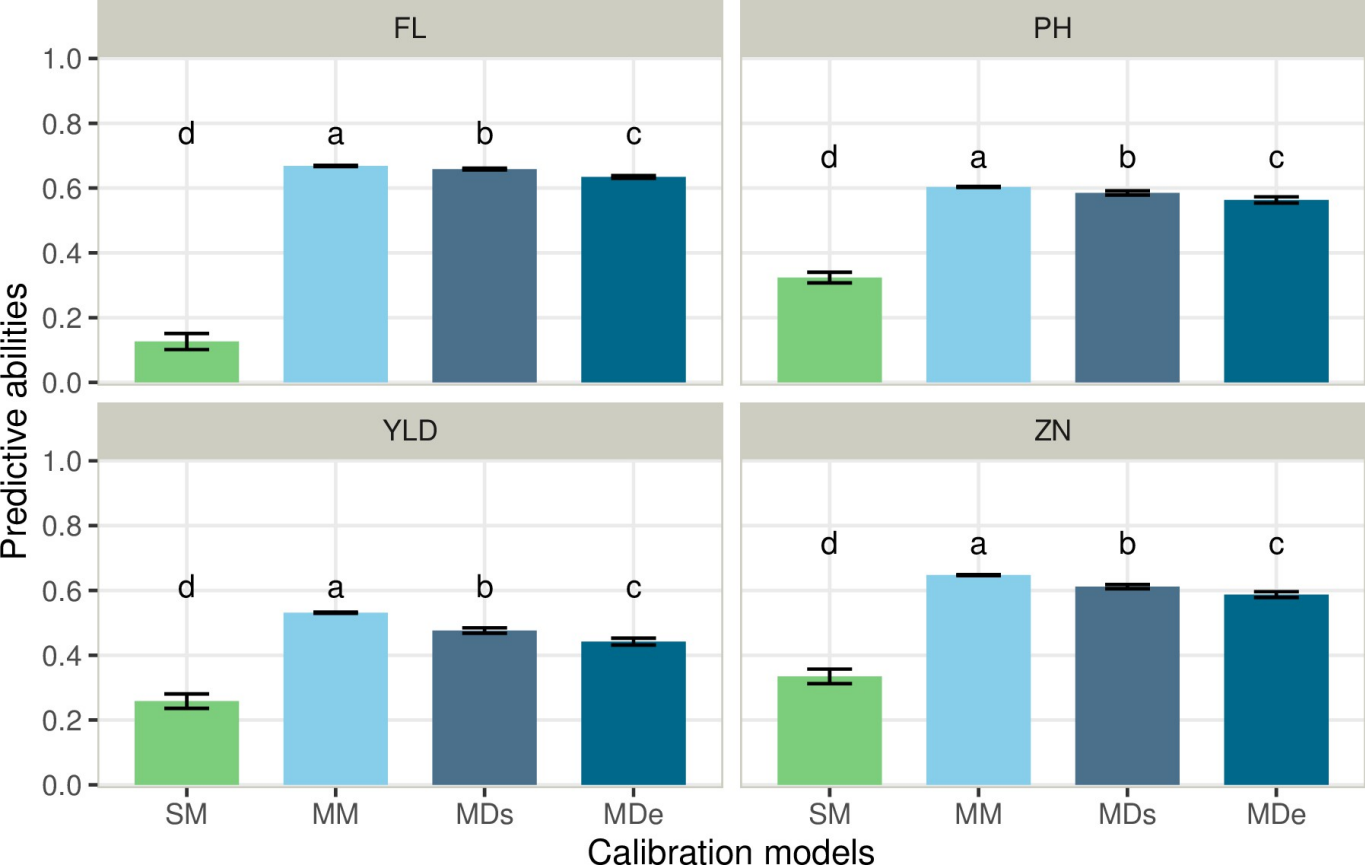
Predictive abilities obtained for the ALU condition using four genomic prediction models. The comparison was done between a single-environment model (SM) and multi-environment models (MM, MDs and MDe, see the definition in the method section) for each of the four traits (flowering time (FL), plant height (PH), grain yield (YLD) and zinc concentration in polished grain (ZN)). The letters in each panel indicate a significant difference between means based on Tukey’s range test.

GS was performed with four models differing in their capacity to account for GxE interactions. In addition to the additive genetic variance, all models included a random intercept for each line to capture residual genetic variation. The model with the lowest proportion of residual variances for all traits was MDe followed by SM (except for FL, Table 4 and S2 Table). The MM model presented the highest residual variances in proportion to the total variance explained by the model. As expected, the genetic variances for the SM and MM models were higher compared to MDs and MDe, as the interaction components explained more than 20% of the total variance (Table 4). For all traits, the performance of the families in ALU was predicted (Fig 3 and Table 3). The model taking only one site into account for the prediction (SM) resulted in lower PAs than multi-environment models for all traits. The PA was the lowest for FL (0.13) and the highest for ZN (0.33). When data from the two conditions were considered to predict performances in ALU, a significant increase in PA was observed for all traits (Fig 3). The increase in PA varied depending on the model, with a range of 71% to 430% for YLD and FL, respectively. The modeling of GxE interaction had a reduced impact on the PA for most of the traits. Indeed, the best model for all traits was the one that only considered the genetic variance and did not include GxE interaction (MM). The best PAs for each trait were observed for the MM model: 0.67, 0.65, 0.60, and 0.53 for FL, ZN, PH, and YLD, respectively. The modeling of an environment-specific variance deviation effect (MDe) resulted in a significant reduction of PA in all the cases compared to the model with a single random deviation effect (MDs).

**Table 4:** Proportion of variance accounted for by each component of the model for the four traits considering the four models. The four models are a single-environment model (SM) and multi-environment models (MM, MDs, and MDe, see the definition in the method section) for each of the four traits: flowering time (FL), plant height (PH), grain yield (YLD) and zinc concentration in polished grain (ZN). The variance components are defined as $\sigma_{\epsilon }^{2}$ for the residual variance, $\sigma_{g}^{2}$ for the genetic variance, $\sigma_{I}^{2}$ for the variance of the random intercept, $\sigma_{ge}^{2}$ for the variance resulting from the GxE interaction, and $\sigma_{ALU}^{2}$ and $\sigma_{LIM}^{2}$ are the two environment-specific variances.

Trait	Variance component	SM_ALU	MM	MDs	MDe
**FL**	$\sigma_{\epsilon }^{2}$	16%	20%	11%	5%
	$\sigma_{g}^{2}$	54%	55%	42%	23%
	$\sigma_{I}^{2}$	30%	25%	22%	13%
	$\sigma_{ge}^{2}$			25%	
	$\sigma_{ALU}^{2}$				23%
	$\sigma_{LIM}^{2}$				37%
**PH**	$\sigma_{\epsilon }^{2}$	11%	23%	12%	6%
	$\sigma_{g}^{2}$	68%	62%	48%	26%
	$\sigma_{I}^{2}$	20%	14%	13%	8%
	$\sigma_{ge}^{2}$			26%	
	$\sigma_{ALU}^{2}$				32%
	$\sigma_{LIM}^{2}$				28%
**YLD**	$\sigma_{\epsilon }^{2}$	13%	28%	16%	8%
	$\sigma_{g}^{2}$	64%	57%	43%	22%
	$\sigma_{I}^{2}$	23%	15%	13%	7%
	$\sigma_{ge}^{2}$			28%	
	$\sigma_{ALU}^{2}$				39%
	$\sigma_{LIM}^{2}$				25%
**ZN**	$\sigma_{\epsilon }^{2}$	11%	23%	13%	7%
	$\sigma_{g}^{2}$	69%	60%	48%	27%
	$\sigma_{I}^{2}$	20%	17%	16%	9%
	$\sigma_{ge}^{2}$			23%	
	$\sigma_{ALU}^{2}$				29%
	$\sigma_{LIM}^{2}$				28%

### Performance of the selected families

**Fig 4:**
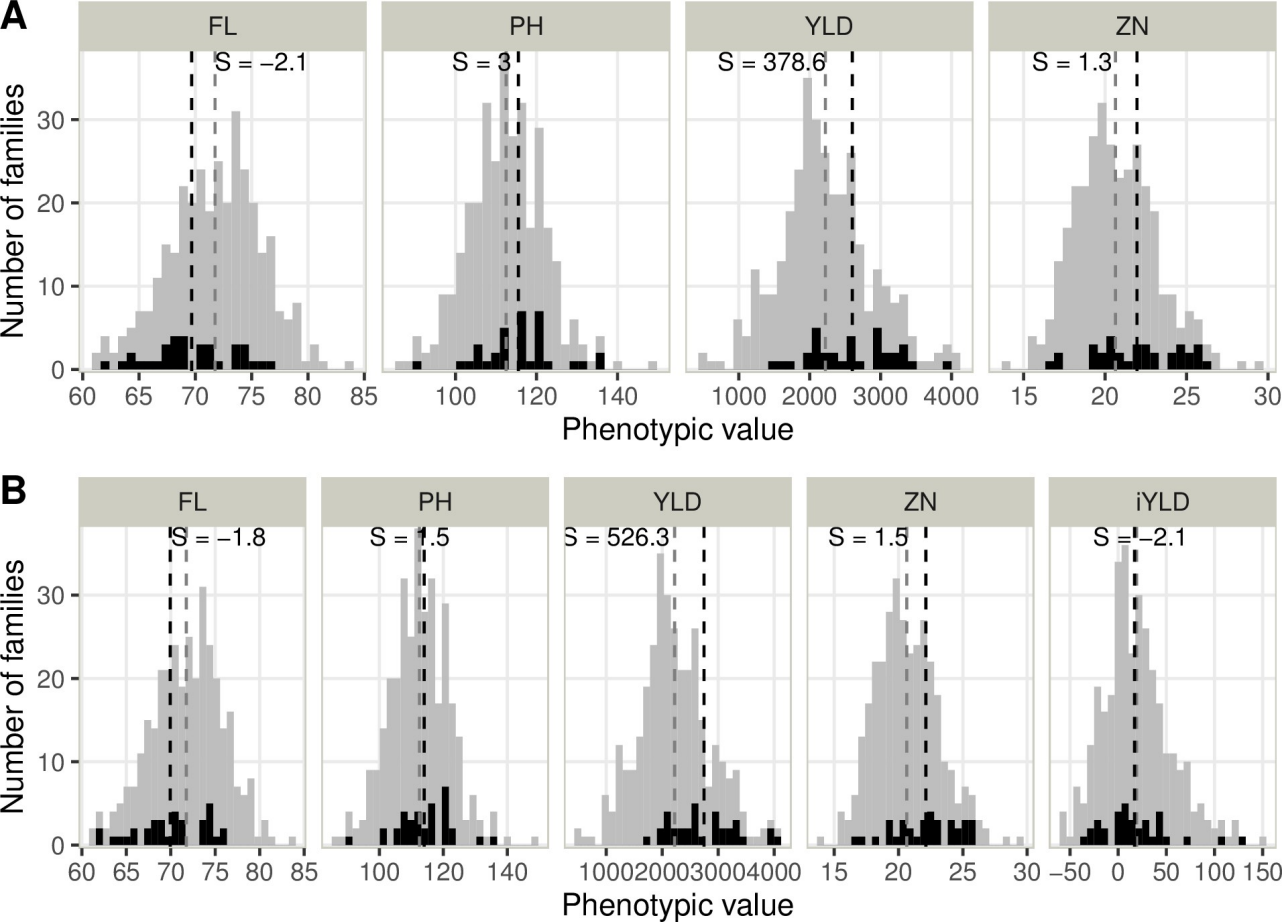
Distribution of the phenotypic value in ALU for the selected families (in black) and the rest of the population (in grey). The selection was based on the GS index (panel A) and phenotypic selection index (panel B). The two indices are defined in the material and methods section. The four traits (flowering time (FL), plant height (PH), grain yield (YLD), and zinc concentration in polished grain (ZN)) and the yield stability index (iYLD) are represented. The vertical dashed lines represent the mean values for the selected families (in black) and the entire population (in grey). The selection differential (S) is indicated for each trait in the trait’s units.

**Fig 5:**
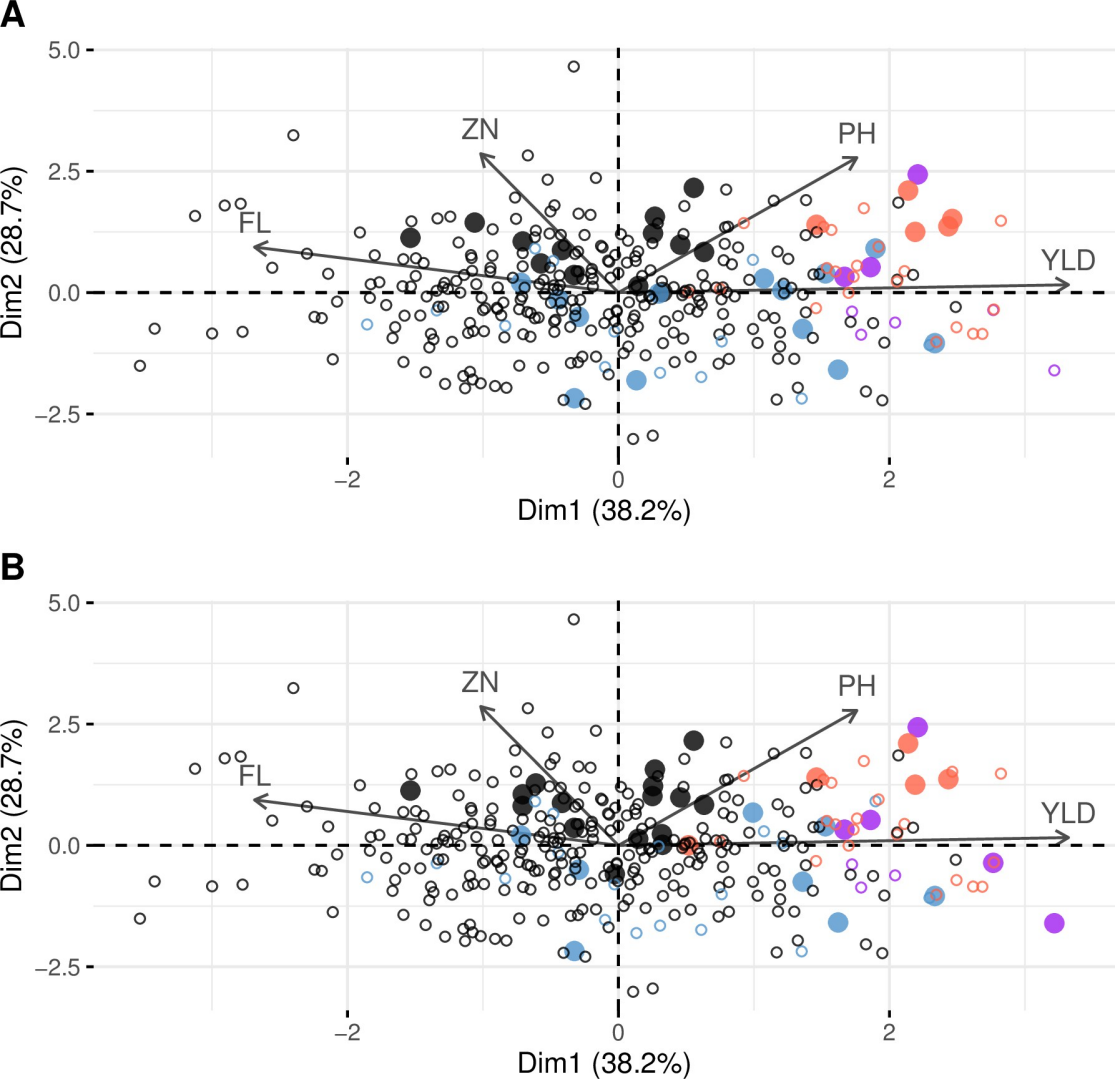
Phenotypic diversity in ALU of the population and the selected families. The families selected with the GS index are shown in panel A, and those selected with the phenotypic selection index are shown in panel B. The population is represented with circles, and the selected families are represented by large dots. The colors represent the different classes of yield performance according to the conditions (see Fig 2). The four traits (flowering time (FL), plant height (PH), grain yield (YLD), and zinc concentration in polished grain (ZN)) and the yield stability index (iYLD) are represented.

The families selected based on the multi-trait index presented, on average, a higher PH, YLD, ZN, and iYLD and a lower FL (Fig 4). Despite the weights to reduce PH and maintain FL, the higher weight on YLD and the correlations with YLD (positive for PH and negative for FL), the selection differential was positive for PH (+ 3 cm with GSi, +1.5 cm with PSi) and negative for FL (- 2.1 days with GSi, -1.8 with PSi). The selection differential was positive for the other traits: + 378.6 kg/ha for YLD, + 1.3 ppm for ZN using GSi and + 526.3 kg/ha for YLD, + 1.5 ppm for ZN using PSi. Integrating iYLD in PSi helped select families with higher yields in ALU and more tolerant families compared to GSi. (Fig 5). Out of the top eight highest-yielding families in the two conditions, five were selected using PSi and three using GSi.

## Discussion

### Upland rice performance under aluminum toxicity

The PCT27 population’s response to Al toxicity indicated good tolerance. On average, the population flowered two days later and was 4 cm taller under acid soil conditions. Interestingly, the average grain yield was greater under the natural acid soil conditions than limed conditions. These results were consistent with the observations made on a population of recurrent selection at the foundation of the synthetic PCT27, reporting greater yield under higher levels of Al toxicity [45]. Similar results were reported by Arbelaez et al. [63], who conducted a study on the adaptation of rice germplasm to upland acid soil conditions. They used field phenotyping to evaluate introgression lines developed from a cross between wild and cultivated rice. While the introgression lines flowered earlier and were smaller in acid soils than in limed conditions, no particular differences were found for traits related to productivity, such as tiller number or panicle number. In controlled conditions, Kang et al. [64] found a delay in flowering time of nine days for two tolerant lines under a low level of Al toxicity. Contrary to our study, they observed a significant drop (up to 42%) in grain yield for tolerant varieties, which was linked to a decrease in the number of panicles per plant and filled grains per panicle. These differences are related to the material studied and the conditions of evaluations of Al toxicity. Most recent studies on rice have been conducted under managed conditions, primarily through hydroponics [64–68]. While evaluations at the vegetative stage in controlled conditions present several advantages compared to field evaluations, limitations can arise in the context of applied breeding. A limited number of studies have compared the level of tolerance obtained under hydroponic conditions and the level of productivity in the field. Howeler & Cadavid [8] found a correlation of 0.64 between grain yield in field conditions and the response to Al toxicity for 240 rice cultivars. The diversity of the material evaluated (with tolerant and susceptible lines) could explain this good correlation. In our study, the population is more homogeneous, as highlighted by the lack of evident genetic structure and the moderate GxE found for all the traits, including grain yield. Indeed, most families (72%) were classified as tolerant with iYLD > 0. This large proportion of families having greater yield under ALU than under LIM indicates that the source population, which was improved for many cycles under savanna soil conditions, may have been under strong selection for Al tolerance, thus holding reduced variability for that trait. Loss of additive variance for adaptive traits is not rare for traits under strong selection, as reported for tropical japonica rice growing in Al stress conditions [69]. In the context of recurrent selection on a japonica population, this reinforces the interest in genomic prediction to better capture minor genetic effects for Al tolerance.

### Genomic selection for tolerance to aluminum toxicity

Several studies have investigated the interest of genomic prediction for rice breeding; however, none have delved into its potential application for the selection of Al toxicity tolerance [28]. While most studies using multi-environment GS have aimed to predict genotype performance and overall stability across various conditions, our objective was to select for adaptation to acid soils. The accuracy of these studies varied greatly depending on several factors, including the trait being studied, the type of population, and the validation method used. Accuracies typically ranged from 0 to 0.80, leading to different interpretations of the added value of GS for breeding programs. In our study, the single-environment model to predict families’ performance under ALU conditions yielded low PAs ranging from 0.13 to 0.33. These PAs were lower than those previously reported for similar genetic material with a comparable training set size [46, 48]. Although the phenotypic variability for each trait was similar between studies, we observed a reduced level of broad-sense heritability in the present experiment that can explain the lower PAs. With PAs ranging from 0.44 to 0.67, multi-environment models outperformed single-environment models for all the traits. Previous studies on rice using a similar cross-validation approach (prediction of unobserved phenotypes of individuals evaluated at least in one environment) have shown that multi-environment models can significantly improve PAs by allowing information sharing across correlated environments [48, 70–72]. However, we observed a decrease in accuracy for most traits when the GxE interactions were included in the models (MDs and MDe). This tendency contrasts with the generally reported benefit of integrating GxE in the prediction models [31, 73]. In a related study on maize and wheat, Cuevas et al. [57] showed that multi-environment models that integrate GxE with or without a specific variance for each environment outperformed other models. They also found that including a random intercept of each line in the model captured extra genetic variability, but only for GBLUP. On rice, Monteverde et al. [72] tested the multienvironment modeling approach for GS, considering three years of phenotypic evaluation to represent the environment parameter. They reported a greater accuracy when including covariance between environments in the model. The proper modeling of genetic correlations between environments had a direct impact on PA. The study found that the multi-environment GBLUP models perform better when an unstructured covariance matrix is used, as compared to when a separate variance component per environment is used, assuming no genetic correlation between environments. The authors emphasized the advantage of incorporating information from multiple environments in the prediction models, as it enables the models to use the correlation between different environments and leverage information across them.

Our multi-environment is limited to ALU and LIM conditions. To improve the population’s tolerance to Al, we predicted the grain yield under ALU, but also considered the index iYLD. The PA for the stability index was very low and much lower than for the quantitative traits from which it is derived. In the context of multi-environment evaluation, Huang et al. [74] reported that prediction accuracy for trait stability was similar to or higher than that for predicting directly phenotypic performance. This finding suggests the potential for selecting for adaptability, especially for traits with high GxE, such as grain yield. However, in the case of tolerance to abiotic stress, predicting stability or susceptibility indices obtained from traits measured in contrasting environments has proven challenging. Cerrudo *et al*. [75] and Ben Hassen *et al*. [70] studied the performance of GS for abiotic stress tolerance and alternative cultivation methods. Cerrudo *et al*. [75] examined drought stress susceptibility in a population of wheat lines. They found that the prediction accuracy for yield under drought conditions was lower than under well-watered conditions due to the reduced phenotypic variation for drought stress susceptibility among the studied population. Similarly, Ben Hassen *et al*. [70] studied the performance of prediction models for rice lines evaluated under alternate wetting and drying and irrigated fields with continuous flooding conditions. The authors used two parameters to test the performance of rice under the two water regimes: the regression slope, which measures stability, and the index of relative performance. The prediction accuracy obtained with the regression slope was low and even lower when using the index. To explain the low prediction accuracy obtained with the index of relative performance, the authors suggested a limited genetic control of variation for the response index. In line with their findings, the predictive ability for iYLD in this study was low. Indeed, whether the index was predicted directly or calculated based on environment predictions, its accuracy was very low. This result highlights the cumulative nature of prediction imprecision and the difficulty of GS models in capturing GxE when summarized into an index.

### Implications for breeding

Genomic prediction is an interesting tool, especially in the context of population improvement through recurrent selection, as it is implemented in the CIAT-CIRAD breeding program. Indeed, it enables a reduction of the breeding cycle length while increasing the selection intensity. Genomic prediction has been shown to be an effective tool to reduce cycle time in rice breeding [28]. It presents several advantages in the case of recurrent selection, as the recycling of the best families can be decoupled from their evaluation thanks to across-cycle prediction. We have previously shown that cycle time can be reduced from three years to one year [46, 48]. In addition, predicting among a set of families much larger than the set used to train the model can increase the selection intensity, especially for traits that require more effort to evaluate. Furthermore, the benefits of using genomic prediction rely on the fact that phenotyping does not have to be performed for the whole population in all the environments of interest. Utilizing shared information from different environments through sparse testing can benefit breeding programs, both in terms of time and cost [76, 77]. In the present case, improving the population for standard growing conditions while maintaining its high tolerance to Al toxicity could be done by using a sparse testing approach and genomic prediction. A subset of families would be evaluated in acid soils and another overlapping subset in normal soil conditions [29].

Our intent was to predict the performance of lines extracted from an unstructured population to select either new parental lines for the next cycle of selection or candidates to enter the pedigree breeding scheme for variety development. The multitrait selection index based on the predicted performance of the families in the stress condition (ALU) while gathering the shared information from the performance in the standard condition (LIM) offered a good compromise to select material with good performance in both conditions. The gain in YLD_ALU achieved using the GS index (GSi) was lower than with the phenotypic selection index (PSi) (17% and 24% gain for GSi and PSi, respectively), and this probably resulted from greater consideration of grain yield parameter in the latter (integration of iYLD). However, the selection process results in terms of selected families demonstrated that the GSi is effective in selecting families with good performance under both ALU and LIM conditions. It is worth noting that most of the information used in creating the GSi was derived from the LIM condition. Selection indices are powerful tools in the context of recurrent selection as they move the population mean across cycles in the desired direction for all traits, even when they are negatively correlated [78]. In our case, yield was negatively correlated with both days to flowering and zinc content in the two conditions. Nonetheless, the selection index allowed the selection of families with a potential to deliver a genetic gain of 0.4 t/ha for grain yield under Al toxic soils, while improving grain zinc concentration by +1.3 ppm. In a recent study on rice, Ramos Guimarães et al. [79] used different selection indices to identify families that could be recombined in a recurrent population. The selection indices included six traits: grain yield, plant height, days to flowering, panicle blast, leaf scald, and grain discoloration. By using these indices, they were able to efficiently select for grain yield while reducing the other traits. The most suitable index was based on the rank of the genotypes [80]. The main advantage of this index is that it does not require the estimation of genetic parameters or the assignment of weights, and so is easy to use. One important aspect of using selection indices is the assignment of meaningful economic weights, which is a major challenge for breeders. In the present study, the weights were assigned based on historical data from the program. In the future, it will be interesting to compare the index derived from the ranking of evaluated families with the one based on weights. If the former performs better, it would be simpler to use it in the breeding programs due to its easy estimation.

## Conclusion

This study shows that a large proportion of the PCT27 population presents tolerance to Al toxicity, with families with high yield in both standard and stress conditions, offering a chance for GS to improve the population further. Applying genomic prediction in the S_0_ generation could significantly speed up the breeding program by decoupling family selection from phenotypic evaluation. Genomic prediction and sparse testing show promise for enhancing crop stability across environments with acid soils while considering GxE interactions. Indeed, our goal in the breeding program is to improve rice’s productive potential under favorable conditions in terms of productivity and grain quality, as well as its tolerance to Al toxicity. The selection index must be carefully defined, considering both overall performance and stability.

## Supporting information

S1 Fig Distribution of the molecular markers used in this study across the rice genome. The number of SNPs on each chromosome is indicated at the bottom.
(PDF)

S2 Fig Distribution of minor allele frequency (MAF) for the molecular markers genotyped on the population. (PDF)

S3 Fig Change in linkage disequilibrium among markers along the 12 chromosomes. (PDF)

S4 Fig Unweighted neighbor-joining tree for all 334 S_0_ genotypes evaluated in the study. The genetic distance was computed as one minus the coefficient of relatedness.
(PDF)

S5 Fig Pearson correlations among the four traits (flowering time (FL), plant height (PH), grain yield (YLD), and zinc concentration in polished grain (ZN)) and the index iYLD for ALU (A) and LIM (B) conditions. The stars after the correlation value indicate the level of significance: * for p-values < 0.05, ** for p-values < 0.01, and *** for p-values <0.001.
(PDF)

S6 FigPearson correlation between observed (iYLD) and predicted (e_iYLD) stability index for grain yield.The i_eYLD is the index calculated from the predicted values of grain yield in the two conditions as $\frac{YLD\_ALU_{SM}-YLD\_LIM_{SM}}{YLD\_LIM_{SM}}*100$. Colored cells are when p-values exceeded 0.05.(PDF)

S1 Table Predictive ability for the four traits (flowering time (FL), plant height (PH), grain yield (YLD), and zinc concentration in polished grain (ZN)) using the single-environment model (SM). The families’ performance was predicted for the two soil conditions: ALU and LIM and 10 replicates for the 5-fold cross-validation were used to estimate the mean and standard deviation (sd).
(PDF)

S2 TableEstimated variance components for the four traits considering the four models.The four models are a single-environment model (SM) and multi-environment models (MM, MDs and MDe, see the definition in the method section) for each of the four traits; flowering time (FL), plant height (PH), grain yield (YLD) and zinc concentration in polished grain (ZN). The variance components are defined as $\sigma_{\epsilon }^{2}$ for the residual variance, $\sigma_{g}^{2}$ for the genetic variance, $\sigma_{I}^{2}$ for the variance of the random intercept, $\sigma_{ge}^{2}$ for the variance resulting from the GxE interaction, and $\sigma_{ALU}^{2}$ and $\sigma_{LIM}^{2}$ are the two environment-specific variances. Standard deviations in parentheses.(PDF)
