# Supplementary material for: Genomic selection for tolerance to aluminum toxicity in a synthetic population of upland rice
Source: PLoS One. 2024 Aug 22;19(8):e0307009. doi: 10.1371/journal.pone.0307009 (PMC11341055; doi:10.1371/journal.pone.0307009)
Supplement: S1 Fig — The number of SNPs on each chromosome is indicated at the bottom. (PDF) [file pone.0307009.s001.pdf]

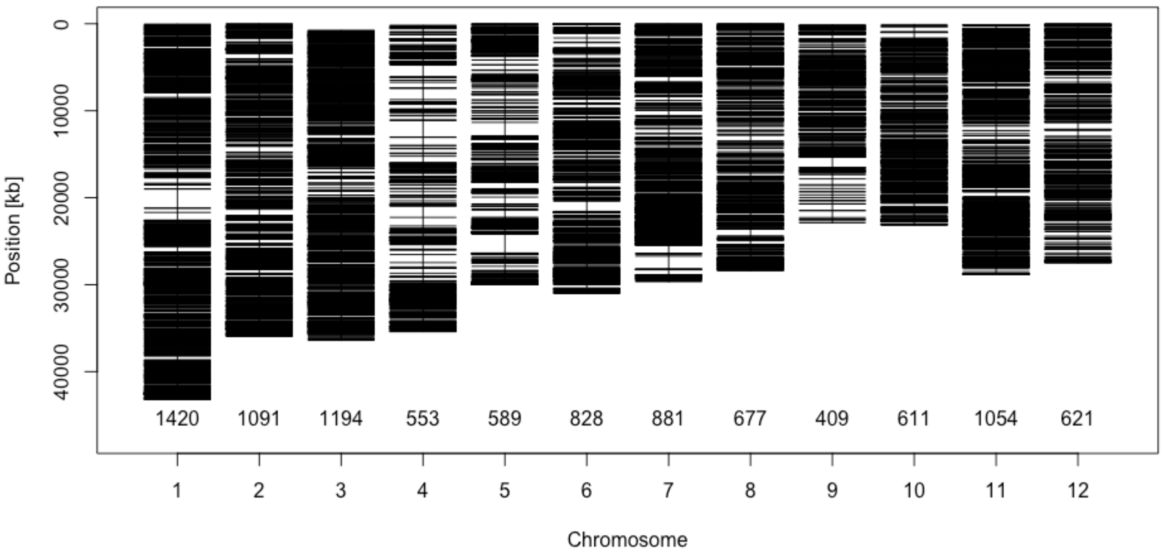

S1 Fig. Distribution of the molecular markers used in this study across the rice genome. The number of SNPs on each chromosome is indicated at the bottom.
