# Supplementary material for: Genomic selection for tolerance to aluminum toxicity in a synthetic population of upland rice
Source: PLoS One. 2024 Aug 22;19(8):e0307009. doi: 10.1371/journal.pone.0307009 (PMC11341055; doi:10.1371/journal.pone.0307009)
Supplement: S2 Fig — (PDF) [file pone.0307009.s002.pdf]

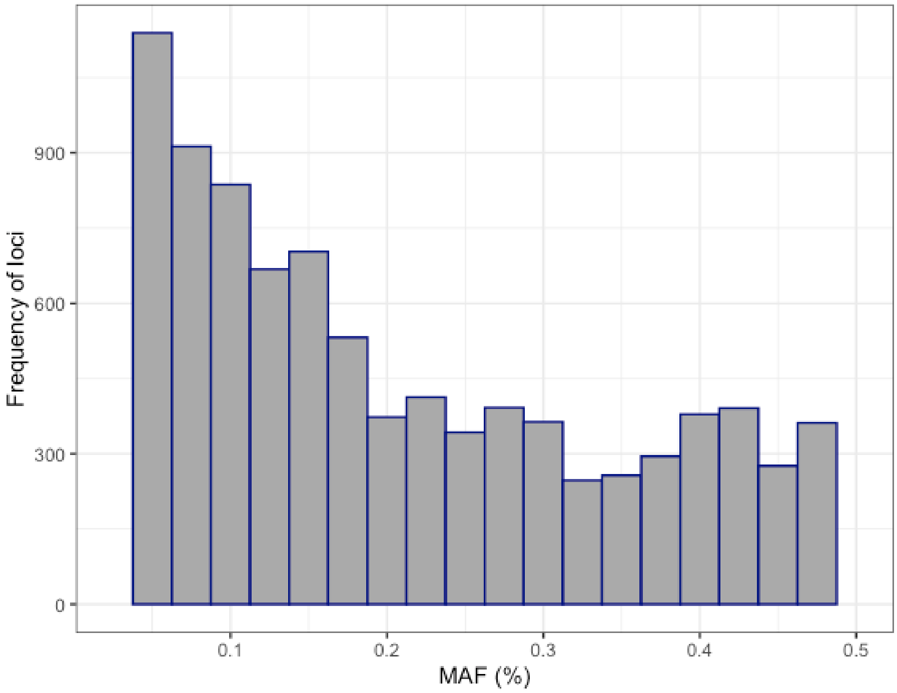

S2 Fig. Distribution of minor allele frequency (MAF) for the molecular markers genotyped on the population.
