# Supplementary figures and images for: Genomic selection for tolerance to aluminum toxicity in a synthetic population of upland rice
Source: PLoS One. 2024 Aug 22;19(8):e0307009. doi: 10.1371/journal.pone.0307009 (PMC11341055; doi:10.1371/journal.pone.0307009)

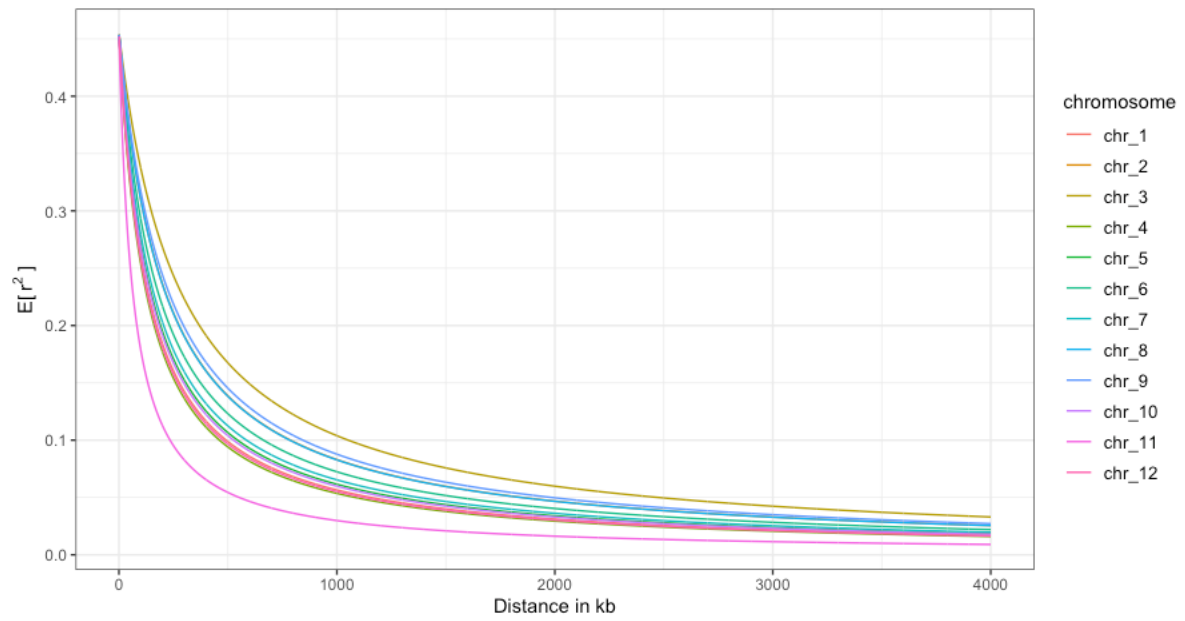

**S3 Fig.** Change in linkage disequilibrium among markers along the 12 chromosomes.

Supplement: S3 Fig — (PDF) [file pone.0307009.s003.pdf]
