# Supplementary material for: Genomic selection for tolerance to aluminum toxicity in a synthetic population of upland rice
Source: PLoS One. 2024 Aug 22;19(8):e0307009. doi: 10.1371/journal.pone.0307009 (PMC11341055; doi:10.1371/journal.pone.0307009)
Supplement: S4 Fig — The genetic distance was computed as one minus the coefficient of relatedness. (PDF) [file pone.0307009.s004.pdf]

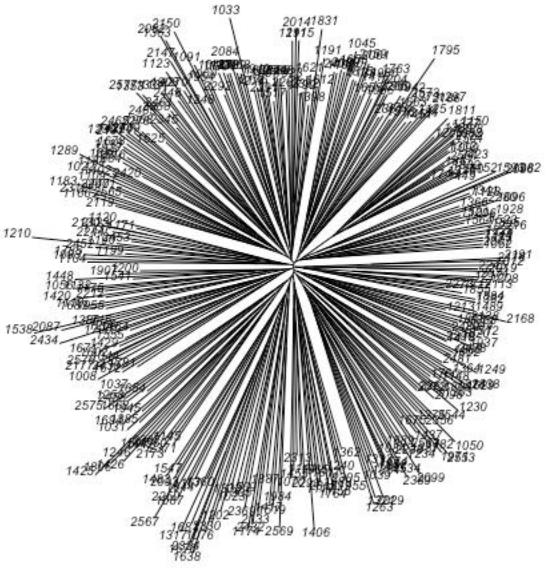

S4 Fig. Unweighted neighbor-joining tree for all 334 S_0 genotypes evaluated in the study. The genetic distance was computed as one minus the coefficient of relatedness.
