# Supplementary material for: Genomic selection for tolerance to aluminum toxicity in a synthetic population of upland rice
Source: PLoS One. 2024 Aug 22;19(8):e0307009. doi: 10.1371/journal.pone.0307009 (PMC11341055; doi:10.1371/journal.pone.0307009)
Supplement: S5 Fig — Pearson correlations among the four traits (flowering time (FL), plant height (PH), grain yield (YLD), and zinc concentration in polished grain (ZN)) and the index iYLD for ALU (A) and LIM (B) conditions. The stars after the correlation value indicate the level of significance: * for p-values < 0.05, ** for p-values < 0.01, and *** for p-values <0.001. (PDF) [file pone.0307009.s005.pdf]

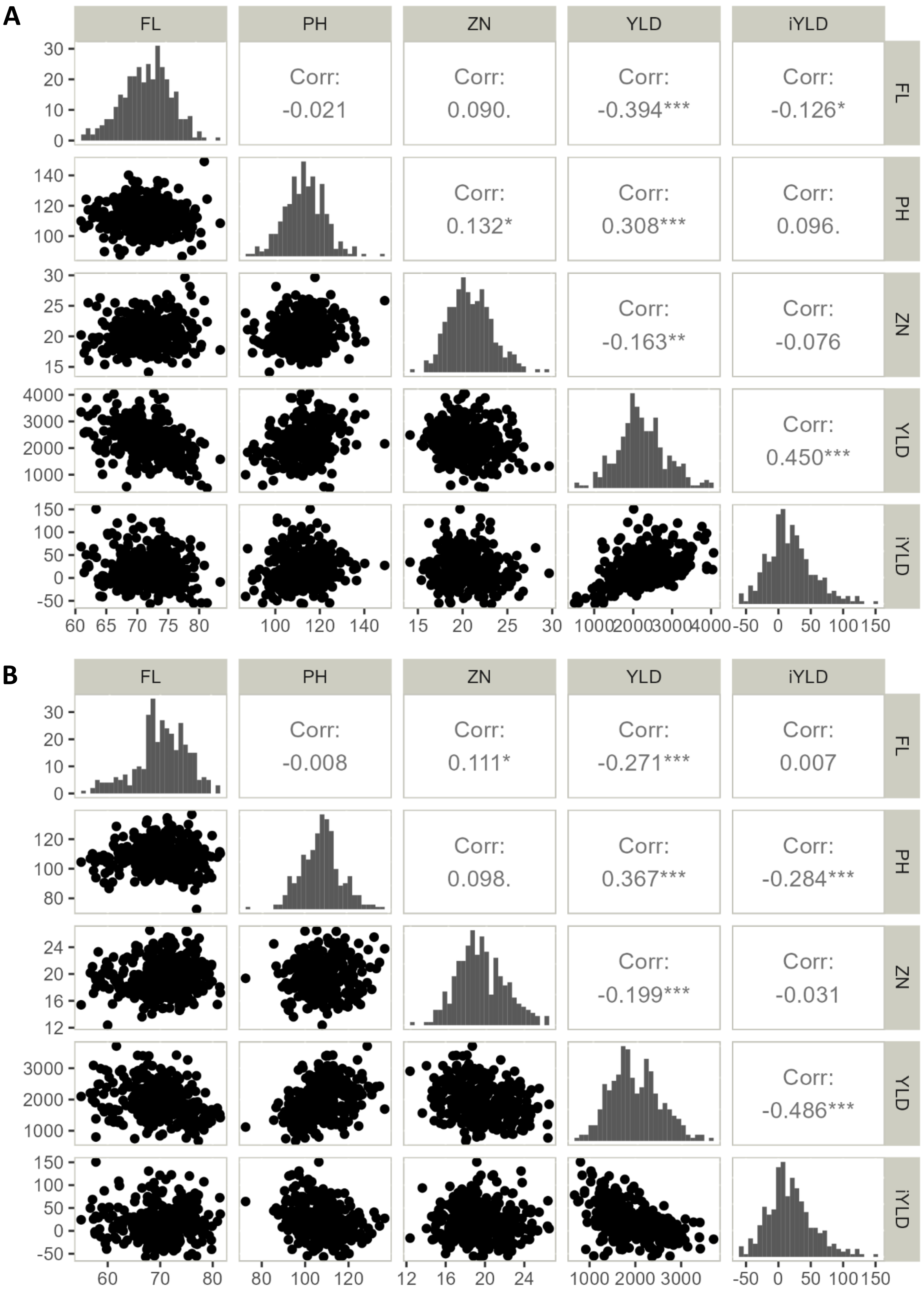

S5 Fig. Pearson correlations among the four traits (flowering time (FL), plant height (PH), grain yield (YLD), and zinc concentration in polished grain (ZN)) and the index iYLD for ALU (A) and LIM (B) conditions. The stars after the correlation value indicate the level of significance: * for p-values < 0.05, ** for p-values < 0.01, and *** for p-values <0.001.
