# Supplementary material for: Genomic selection for tolerance to aluminum toxicity in a synthetic population of upland rice
Source: PLoS One. 2024 Aug 22;19(8):e0307009. doi: 10.1371/journal.pone.0307009 (PMC11341055; doi:10.1371/journal.pone.0307009)
Supplement: S6 Fig — The i_eYLD is the index calculated from the predicted values of grain yield in the two conditions as YLD_ALUSM−YLD_LIMSMYLD_LIMSM*100. Colored cells are when p-values exceeded 0.05. (PDF) [file pone.0307009.s006.pdf]

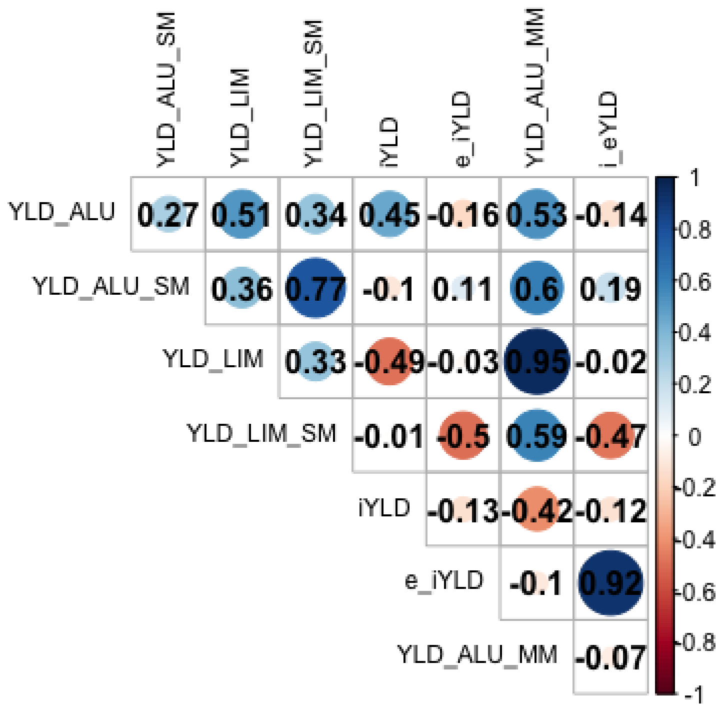

S6 Fig. Pearson correlation between observed (iYLD) and predicted (e_iYLD) stability index for grain yield. The i_eYLD is the index calculated from the predicted values of grain yield in the two conditions as $\frac{YLD\_ALU_{SM} - YLD\_LIM_{SM}}{YLD\_LIM_{SM}} * 100$. Colored cells are when p-values exceeded 0.05.
