# Supplementary material for: Genomic selection for tolerance to aluminum toxicity in a synthetic population of upland rice
Source: PLoS One. 2024 Aug 22;19(8):e0307009. doi: 10.1371/journal.pone.0307009 (PMC11341055; doi:10.1371/journal.pone.0307009)
Supplement: S1 Table — The families’ performance was predicted for the two soil conditions: ALU and LIM and 10 replicates for the 5-fold cross-validation were used to estimate the mean and standard deviation (sd). (PDF) [file pone.0307009.s007.pdf]

S1 Table. Predictive ability for the four traits (flowering time (FL), plant height (PH), grain yield (YLD), and zinc concentration in polished grain (ZN)) using the single-environment model (SM). The families' performance was predicted for the two soil conditions: ALU and LIM and 10 replicates for the 5-fold cross-validation were used to estimate the mean and standard deviation (sd).

	SM_ALU		SM_LIM	
Trait	mean	sd	mean	sd
FL	0.125	0.025	0.23	0.028
PH	0.324	0.029	0.361	0.022
YLD	0.258	0.031	0.317	0.018
ZN	0.343	0.032	0.225	0.038
