# Supplementary material for: Genomic selection for tolerance to aluminum toxicity in a synthetic population of upland rice
Source: PLoS One. 2024 Aug 22;19(8):e0307009. doi: 10.1371/journal.pone.0307009 (PMC11341055; doi:10.1371/journal.pone.0307009)
Supplement: S2 Table — The four models are a single-environment model (SM) and multi-environment models (MM, MDs and MDe, see the definition in the method section) for each of the four traits; flowering time (FL), plant height (PH), grain yield (YLD) and zinc concentration in polished grain (ZN). The variance components are defined as σϵ2 for the residual variance, σg2 for the genetic variance, σI2 for the variance of the random intercept, σge2 for the variance resulting from the GxE interaction, and σALU2 and σLIM2 are the two environment-specific variances. Standard deviations in parentheses. (PDF) [file pone.0307009.s008.pdf]

S2 Table. Estimated variance components for the four traits considering the four models. The four models are a single-environment model (SM) and multi-environment models (MM, MDs and MDe, see the definition in the method section) for each of the four traits; flowering time (FL), plant height (PH), grain yield (YLD) and zinc concentration in polished grain (ZN). The variance components are defined as σ_ϵ^2 for the residual variance, σ_g^2 for the genetic variance, σ_I^2 for the variance of the random intercept, σ_{ge}^2 for the variance resulting from the GxE interaction, and σ_{ALU}^2 and σ_{LIM}^2 are the two environment-specific variances. Standard deviations in parentheses.

Trait	Variance component	SM_ALU	MM	MDs	MDe
**FL**	σ_ϵ^2	4.94	8.47	5.30	4.38
		(1.77)	(0.76)	(0.66)	(0.66)
	σ_g^2	16.77	23.08	21.43	19.95
		(4.92)	(5.81)	(5.66)	(5.31)
	σ_I^2	9.21	10.61	11.52	11.18
		(2.00)	(1.55)	(1.52)	(1.48)
	σ_{ge}^2			13.08	
				(2.71)	
	σ_{ALU}^2				20.45
					(4.11)
	σ_{LIM}^2				32.55
					(7.18)
**PH**	σ_ϵ^2	21.71	42.74	26.73	23.17
		(7.43)	(3.55)	(3.08)	(3.10)
	σ_g^2	127.92	113.87	104.67	96.11
		(36.34)	(24.31)	(24.67)	(23.84)
	σ_I^2	40.05	26.68	29.50	29.58
		(8.82)	(4.66)	(4.77)	(4.70)
	σ_{ge}^2			58.54	
				(12.81)	
	σ_{ALU}^2				120.94
					(27.10)
	σ_{LIM}^2				104.88
					(21.97)
**YLD**	σ_ϵ^2	108570.35	195913.66	137114.84	117274.68
		(37620.49)	(15841.61)	(14735.18)	(15024.91)
	σ_g^2	542477.31	403304.75	372045.83	340860.66
		(156833.43)	(87045.90)	(88001.63)	(83940.60)
	σ_I^2	200934.84	105656.11	114017.76	114063.98
		(43891.62)	(18334.71)	(18649.52)	(18359.66)
	σ_{ge}^2			243136.15	
				(55218.95)	
	σ_{ALU}^2				626342.71
					(151456.75)
	σ_{LIM}^2				393333.34
					(81179.06)
**ZN**	σ_ϵ^2	1.46	2.92	1.94	1.71
		(0.50)	(0.25)	(0.22)	(0.22)
	σ_g^2	9.13	7.68	7.22	6.70
		(2.55)	(1.75)	(1.77)	(1.70)
	σ_I^2	2.69	2.22	2.45	2.45

Trait	Variance component	SM_ALU	MM	MDs	MDe
		(0.59)	(0.38)	(0.38)	(0.38)
	σ_{ge}^2			3.57	
				(0.77)	
	σ_{ALU}^2				7.60
					(1.65)
	σ_{LIM}^2				7.05
					(1.51)
